# Pharmacists’ involvement in COVID-19 vaccination across Europe: a situational analysis of current practice and policy

**DOI:** 10.1007/s11096-021-01301-7

**Published:** 2021-07-03

**Authors:** Vibhu Paudyal, Daniela Fialová, Martin C. Henman, Ankie Hazen, Betul Okuyan, Monika Lutters, Cathal Cadogan, Filipa Alves da Costa, Elena Galfrascoli, Yvonne Marina Pudritz, Silas Rydant, Jaime Acosta-Gómez

**Affiliations:** 1 School of Pharmacy, College of Medical and Dental Sciences, University of Birmingham, Edgbaston, Birmingham, B15 2TT UK; 2 Department of Social and Clinical Pharmacy, Faculty of Pharmacy in Hradec Králové, Charles University, Prague, Czech Republic; 3 Department of Geriatrics and Gerontology, 1st Faculty of Medicine in Prague, Charles University, Prague, Czech Republic; 4 School of Pharmacy and Pharmaceutical Sciences, Trinity College Dublin, Dublin, Ireland; 5 Centre for Pharmacy Postgraduate Education, University of Manchester, Manchester, UK; 6 Clinical Pharmacy Department, Faculty of Pharmacy, Marmara University, Istanbul, Turkey; 7 Clinical Pharmacy, Cantonal Hospital Baden, Baden, Switzerland; 8 Swiss Federal Institute of Technology, Zurich, Switzerland; 9 Faculty of Pharmacy, University of Lisbon, Lisbon, Portugal; 10 Hospital Pharmacy, ASST Settelaghi, Varese, Italy; 11 LMU Hospital Pharmacy, Marchioninistr, 15, 81377 Munich, Germany; 12 Meduca, Koninklijke Apothekersvereniging Antwerpen (KAVA), Lange Leemstraat 187, 2018 Antwerpen, Belgium; 13 Farmácia Acosta, Calle Vitoria, 9, 28941 Fuenlabrada, Madrid, Spain

**Keywords:** Coronavirus, COVID-19, Pharmacist, Pharmacy, Public health, Vaccine

## Abstract

One year since the emergence of the COVID-19 pandemic, rapid response measures have been implemented internationally to mitigate the spread of the virus. Following rapid and successful pre-clinical and human trials, several vaccines have been authorised for use across Europe through the European Medicines Agency and national regulatory authorities. Clinical trials have shown promising results including important reductions in disease severity, hospitalisation and mortality. In order to maximise the public health benefit of available vaccines, there is a pressing need to vaccinate a large proportion of the population. Internationally, this has prompted coordination of existing services at enormous scale, and development and implementation of novel vaccination strategies to ensure maximum inoculation over the shortest possible timeframe. Pharmacists are being promoted as healthcare professionals that enhance roll-out of COVID-19 vaccination programmes. This paper aims to summarise current policy and practice in relation to pharmacists’ involvement in COVID-19 vaccination in 13 countries across Europe.

## Introduction

In March 2020, the COVID-19 outbreak was declared a global pandemic [1]. Since the first cluster of cases was detected in the Wuhan province of China in December 2019, COVID-19 has been responsible for over three million deaths globally, almost one third of which have occurred in Europe [2]. Following rapid and successful pre-clinical and human trials, several vaccines have been developed by partnerships including Astra Zeneca/Oxford University, Pfizer/BioNTech, Moderna. These vaccines have since been authorised for clinical use by the European Medicines Agency (EMA) and some national drug regulatory authorities [3]. For the EMA-approved vaccines, clinical trials have shown promising results in terms of reductions in COVID-19 disease severity, hospitalisation and mortality [4].

While the development of effective vaccines provides an important means of reducing the public health threat posed by COVID-19, vaccination uptake rates of at least 67 % are needed at a population level [5]. Internationally, this has prompted coordination of existing services at enormous scale [6–8], and development and implementation of novel vaccination strategies to ensure maximum inoculation of the population in the shortest possible time frame [9–11]. A study demonstrated pharmacists’ diverse contributions during the COVID-19 pandemic such as their involvement in clinical care of COVID-19 patients, facilitation of clinical trials, information sourcing and appraisal for promoting evidence-based practice, educating members of public and provision of routine clinical services [12]. In the same study, pharmacists from 16 European countries also demonstrated readiness to be involved in the vaccination process once vaccines were developed and approved [12].

Pharmacists in several European countries have been historically involved in the supply and administration of various vaccines such as influenza, human papilloma viruses and pneumococcal. Given their expertise, clinical educational background and patient-facing roles in diverse settings, professional societies of pharmacists in many countries have been lobbying governments and public health agencies to ensure pharmacists’ inclusion in their COVID-19 vaccination plans and thereby allowing rapid vaccinations in the populations. A previous systematic review has shown that pharmacist-led vaccination programmes are widely accepted patients and help improve the access and rate of vaccination [13]. Political and organisational barriers however, often prevent pharmacists’ participation in the vaccination programmes [13].

To date, there is a lack of comprehensive information describing pharmacists’ involvement in COVID-19 vaccination at international level. This commentary provides a situational analysis of current practices including services and legal framework regarding pharmacists’ involvement in COVID-19 vaccination in 13 countries in Europe. The information in this paper reflects practices and policies up to June 2021 and was gathered and/or written by pharmacy practitioners, academics and relevant experts in respective countries. Legal framework, service specifications and grey reports were consulted where needed. A summary of the situational analysis is presented in Table 1.

**Table 1:** Vaccination against COVID-19 infection in different European countries and involvement of pharmacy services: legal framework, management, provision of vaccination

	Country												
	Belgum	Croatia	Czech Republic	Germany	Italy	Netherlands	Portugal	Ireland	Serbia	Switzerland	Spain	Turkey	UK
*COVID-19 vaccination: legal framework, management, pharmacy services *													
*Legal framework allows vaccinating by*													
Physicians	×	×	×	×	×	×	×	×	×	×	×	×	×
Nurses (under legal responsibility of a physician)	×	×	×	×	×	×			×	×	×	×	×
Nurses independently							×	×					×
Pharmacists (under legal responsibility of a physician)													×
Pharmacists independently				(×) pilots	(×) pilots		×	×		×^d^			×
*Vaccines approved for clinical use in a particular country (considering decision of country-specific regulatory drug institutes by mid of April 2021*)													
Pfizer/BioNTech	×	×	×	×	×	×	×	×	×	×	×	×	×
Moderna	×	×	×	×	×	×	×	×		×	×		×
Astra Zeneca	×	×	×	×	×	×	×	×	×		×		×
Johnson&Johnson	×	×	×	×	×	×	×	×		×	×		×
Sputnik V									×				
Sinovac												×	
*Sinopharm*									×				
*Management of vaccination*													
Central (usually registration at the Ministry of Health website)	×	×	×	×	×	×	×	×^a^	×	×	×	×	×
Different in Federal States/Cantons	×			×	×					×			×
Managed also by individual/group orders at local vacccination centres			×						×		×		×
*Vaccination provided where*													
Large regional hospitals	×		×	×	×	×	×	×		×	×	×	×
Nursing home facilities, homes for seniors	×	×	×	×	×	×	×	×	×	×	×	×	×
Primary health care centres, GP ambulances		×	×	×	×	×	×	×	×	×	×	×	×
Large community centres such as football stadiums, fair trade centres etc.^b^	×	×	×	×	×	×		×	×		×		×
Patients´ own homes (home visits by GPs)	×		×	× (pilot)	×	×	×	×				×	×
Community pharmacies	× (pilot planned)				×			×		×			×
*Community pharmacies/pharmacists - services related to COVID-19 vaccination*													
Administration of vaccines	× (pilot planned)							×		×	×		×
Supply of vaccines to vaccination centres													×
The storage and stock management of vaccines	× (pilot planned)				× (pilot)								×
Preparation (disolution) of vaccines for vaccination centres						×							×
Disolution of vaccines directly at vaccination centres	×				× (pilot)	×							×
Standard practices, safety warnings, dissemination of updates on vaccination procedures etc. (for healthcare professionals at vaccination centres)	×					×	×						×
Counselling (including online counselling) for patients	×	×	×		×	×	×	×		×	×	×	×
Pharmacovigilance activities	×	×	×		×	×	×	×	×	×	×		×
Monitoring and periodic administrative reports	×						×	×					×
*Hospital pharmacies/pharmacists - services related to COVID-19 vaccination*													
Administration of vacccines													×
Management of supply of vaccines to vaccination centres	×		×		×	×	×	×		×	×		×
Storage and stock management of vaccines	×		×	×	×	×	×	×		×	×		×
Preparation (disolution) of vaccines and their delivery to vaccination centres			×	×	×	×	×	×			×		×
Helps with disolution of vaccines directly at vaccination centres			×	×		×	×			×^c^			×
Standard practices, safety warnings, dissemination of updates on vaccination procedures etc. (for healthcare professional, hospital wards)	×		×	×	×	×	×	×		×			×
Counselling (including online counselling) for patients			×		×	×	×	×			×		×
Pharmacovigilance activities	×	×	×		×	×	×	×		×	×		×
Monitoring and periodic adminstrationreports			×		×		×			×	×		×
*Remuneration of vaccines*													
Vaccines fully covered from health insurance/by government for prioritized groups	×	×	×	×	×	×	×	×	×	×	×	×	×

Information correct as of June 2021

*UK* United Kingdom

^a^For priority groups registration is not needed

^b^There are substantial differences among countries which places are used for provision of COVID-19 vaccination: e.g. in Germany also ice skating rinks, former military barracks, university halls, exhibition halls etc.; in the Czech Republic only one Open Trade Fair centre was established as a vaccination centre under managementof regional hospital; in Netherlands also large conference centres are used etc.

^c,d^In some Cantons

## Belgium

In Belgium, COVID-19 vaccinations have been rolled out on a phased basis, starting with residents and staff in residential care facilities and hospital staff; followed by ambulatory healthcare professionals and the general population. Throughout all three phases, pharmacists are responsible for vaccine storage, stock management and preparation in either hospital settings or community vaccination centres. These vaccination centres will close in October 2021 and ambulatory care will be involved afterwards, yet no definitive strategy had been defined. In Belgium, pharmacists are involved in advocacy and promotional activities for all vaccines, including COVID-19, but are legally not allowed to administer vaccines. As of June 2021, both the Belgian government and professional societies however are taking legislative and implementation initiatives (e.g. vaccination courses) to involve community pharmacists in the distribution of COVID-19 vaccines in ambulatory care as well as vaccinator.

## Croatia

In the Republic of Croatia, legal provisions are not currently in place for pharmacists to vaccinate patients. A recent campaign to support influenza vaccination in community pharmacies involved medical doctors vaccinating patients in pharmacies. This campaign sought to convince the government to make vaccination services more accessible to the public through pharmacies [14]. The Croatian Chamber of Pharmacists is working on legislative amendments to enable pharmacists’ involvement in vaccine provision.

To support future involvement of pharmacists in vaccinations, the pharmacy curriculum in Croatia was recently adapted in 2021 to include a new obligatory course “Vaccination in Pharmacy Practice” (for pharmacists that completed their studies before 2021 this course as an elective). Pharmacists have also been involved in manufacturing hand sanitizers in community pharmacies, online patient counselling, and home delivery of medicines during lockdown [15, 16]. None of the services, however, are remunerated by the government.

### Czech Republic

Currently in the Czech Republic, vaccine administration cannot be undertaken by pharmacists. According to the Decree of the Ministry of Health No. 92/2012 Coll [17], based on the minimum technical and material equipment of the health care facilities and contact centres and its annexes, pharmacies were not deemed to be technically ready for vaccination. The current pharmacy undergraduate curriculum does not cover any practical course of vaccination. According to Czech law, vaccine administration (including COVID-19 vaccines) can only be undertaken by physicians, and in the clinical practice is provided also by nurses under the supervision of a physician. Therefore, currently pharmacists can only offer support with COVID-19 vaccines in terms of storage and dispensing. Pharmacists have also been supporting vaccination promotion campaigns held by the Czech Chamber of Pharmacists [18]. The role of hospital pharmacists is similar as in other countries, namely in maintaining standard operating procedures relating to storage and handling of vaccines and in taking responsibility for safe transfer and delivery of vaccines to the vaccination points.

## Germany

In an initial draft for the vaccination rollout, the German Health Ministry recommended pharmacists’ inclusion as mandatory personnel in COVID-19 vaccination centres alongside medical doctors and security personnel [19]. However, in the final draft, pharmacists were no longer listed, and the organisation of these hubs was delegated to the Federal States [20]. Hence, organisation of these hubs differs across each State of Germany. Pharmacists’ current involvement in COVID-19 vaccine roll-out is limited to vaccine logistics, and preparation and they are not allowed to directly administer the vaccines [21]. COVID-19-vaccines are sourced centrally by the government and distributed to logistics hubs which in turn deliver the vaccines locally. From April 2021, general practitioners are being allowed to join the COVID-19 vaccine rollout-out effort, while vaccine supply will be coordinated through community pharmacies and wholesalers [22].

COVID-19-related information has been developed by the Federal Union of German Associations of Pharmacists or the hospital pharmacy association and disseminated via websites, webinars or mailing lists. During the pandemic, hospital and community pharmacies received permission to manufacture medicines and medicinal products that were in short supply due to global manufacturing problems, e.g. disinfectants or midazolam injections [23]. Community pharmacies are involved in supplying and providing rapid COVID-tests and FFP2 masks which are reimbursed at various rates [24]. In addition, pharmacists have been involved in clinical trials relevant to COVID-19 vaccines and drugs [25].

## Italy

The Italian vaccination plan was developed by the Ministry of Health in agreement with the Extraordinary Commissioner for the COVID-19 Emergency in 2020. Within the strategic vaccination plan, at a regional level, hospital pharmacies were chosen as hub to ensure integrity of the COVID-19 vaccine management process. In every hospital hub, lead hospital pharmacists are responsible for the management of the vaccine and for ensuring the handling, storage, transfer of the vaccines from the hub to the local vaccination centres and to the mobile vaccination teams who vaccinate people in long-term care institutions. In addition, they are responsible for ensuring compliance with the regulatory framework under which the vaccines are being administered. Hospital pharmacists develop and maintain standard operating procedures relating to storage and handling and are responsible for the transfer and allocation of doses to territorial vaccination points.

Since December 2020, the Italian Society of Hospital Pharmacists and Italian Society of Compounding Pharmacists have shared joint operating instructions about preparation of doses of COVID-19 vaccines in ready-to-dose syringes. Such instructions were developed on the basis of the European Public Assessment Reports and the Summary of Product Characteristics in order to facilitate and help the healthcare professionals operating in the vaccination sites. They are also in charge of the pharmacovigilance activities, ensuring at the same time consistency and quality of reports and up-to-dated data. Until March 2021, there had been no formal involvement of community pharmacies in administering COVID-19 vaccines. From April 2021, with the progression of the vaccination campaign, the Health Minister signed a protocol to allow community pharmacists qualified by a specific central training course to administer COVID-19 vaccines in their pharmacies helping general practitioners at local level. From June 2021, after completing the required training course, community pharmacists can be involved in the administration of vaccines. However, community pharmacies must guarantee three areas: a reception area, an administration area and a monitoring area. Only the vaccines produced by Johnson & Johnson and AstraZeneca can be administered in a community pharmacy.

## Netherlands

Currently, Dutch pharmacists are legally not allowed to administer vaccines. It is a reserved act according to the Dutch law- Professions in Individual Health Care. Under this law, only the doctors, advanced nurses and physician assistants are considered independently competent’ to administer vaccines by injection. The National Institute for Public Health and the Environment is the lead organisation for the national roll-out of the COVID-19 vaccination in the Netherlands. The vaccines are administered in central hubs run by the municipal health service, in general practices and in care homes. Dutch pharmacists are not currently allowed to administer vaccines. During the COVID-19 vaccine roll-out, both community and hospital pharmacists have played an active role locally in the storage, stock management and preparation of the vaccine and inpatient education. Hospital pharmacies within the national acute care network are responsible for leading the responsibility for receipt, storage and distribution of the vaccine to care homes. Pharmacists have been asked to contact their local health service if they are willing to contribute to the vaccination service.

Guidance and protocols about the different available COVID-19 vaccines and their preparation have been developed for community pharmacists by the Royal Dutch Pharmaceutical Society and for hospital pharmacists by the Dutch Hospital Pharmacists’ Association. The pharmaceutical society also regularly publishes examples of pharmacist’s collaborative initiatives to improve the vaccine role out locally.

## Portugal

Since 2007, community pharmacists in Portugal have been recognised as legal providers of vaccinations which are not covered by the National Health Plan. The service is not yet reimbursed by the Government and is paid out-of-pocket by patients. Pharmacists have therefore expressed their willingness to participate in COVID-19 vaccination. The responsible body for the vaccination strategy has welcomed the availability of pharmacists and expressed the intention of involving pharmacists in future phases of vaccine roll-out. As of June 2021, this has not yet implemented.

## Republic of Ireland

Over the decade preceding COVID-19, pharmacists in the Republic of Ireland have had an increasing role in vaccine provision starting with seasonal influenza vaccination in 2011 [26]. This role has since been extended to include pneumococcal and herpes zoster vaccinations. Legislative changes have been implemented to facilitate continued vaccination provision by pharmacists during the ongoing pandemic [27, 28]. These include provisions for pharmacists to administer COVID-19 vaccines and to vaccinate people outside of the pharmacy setting. However, until the beginning of May 2021, an adequate supply and roll-out of COVID-19 vaccinations in Ireland was an ongoing challenge.

The Department of Health in Ireland published the COVID-19 Vaccination Strategy and Implementation Plan in December 2020 [29, 30] which outlined a three-phase roll-out. The initial phase, where vaccine supply is limited, was to focus on vaccinating the highest priority groups at specified settings, such as long-term care facilities and large-scale healthcare sites. The subsequent phases (ramp-up, open-access), whereby larger numbers of vaccine doses become available, were to involve mass vaccination centres, general practices and community pharmacies. Despite administration of the first COVID-19 vaccine in Ireland in late December 2020, there was no formal involvement of community pharmacies in administering these vaccines until from June 14th, 2021 [31]. The rate to be paid for vaccinations administered by in community pharmacies is set at €25 per dose i.e. €50 per patient where two doses are given alongside an administration fee of €10 per vaccinated patient [32].

## Republic of Serbia

Currently in Serbia, COVID-19 vaccinations are performed by nurses at vaccination centres and at the primary health care centres. There is a lack of policy initiative to promote pharmacists’ involvement in COVID-19 vaccination. A new proposal submitted by pharmacists’ professional society in Serbia was sent to the Ministry of Health in October 2020. This proposal included changes to the community pharmacy services, and among others, it included seasonal influenza vaccination by pharmacists. However, this proposal was rejected by the Government in February 2021. A comparative study on pharmacists´ role and safety practices during the COVID-19 pandemic in Croatia and Serbia was published in 2021 [15]. Currently, a study investigating pharmacists’ readiness to participate in COVID-19 vaccination roll out is being undertaken. Alongside Serbia this study also includes involvement of pharmacists from Romania and Bulgaria.

## Switzerland

In Switzerland, there are 26 Cantons and each Canton is responsible for its own vaccination strategy. Since 2015, community pharmacists of some cantons are allowed to administer vaccines after having passed a specialised training certified by the Swiss Pharmacists Association. Pharmacies are reimbursed 24.50 CHF per vaccination (~ 22.47 Euros). The number of pharmacies involved in vaccination are increasing constantly. In some cantons, pharmacists can administer any approved vaccine, whereas in other cantons, vaccination is restricted to influenza and tick-borne encephalitis.

All COVID-19 vaccines are distributed by the Swiss Army pharmacy to the Cantons that in turn distribute further according to their vaccination strategy. In some Cantons, pharmacies are part of the strategy, while in other Cantons pharmacies are not involved. For example, In the Canton Aargau, the two main Cantonal hospitals, including the hospital pharmacies, had an important role in organizing the first vaccination centres. The hospital pharmacies are responsible for storing and providing the vaccine to the hospital’s vaccination centres, as well as supporting the mobile vaccination teams who vaccinate people in nursing homes and other long-term care institutions. On 1st of March, 2021, Aargovian pharmacists were authorised by the Cantonal government to vaccinate against COVID-19. However, practice has been limited due to supply issues. In other Cantons, a similar situation persists. Pharmacies in Switzerland are also offering assistance in registering patients with a vaccination centre, especially to those who have no access to the internet or who need technical support.

## Spain

Currently pharmacists’ participation in COVID-19 vaccination in Spain is limited. The responsible body for the vaccination strategy is nominated by the Government. The President of the Pharmaceutical Society has publicly expressed the willingness of pharmacists to become involved in the vaccination process. While hospital pharmacists have assisted physicians and nurses in the vaccination process, community pharmacists currently have limited involvement. While hospital pharmacists have assisted physicians and nurses in the vaccination process, community pharmacists currently have limited involvement. Community pharmacy workforce in Madrid has been vaccinated against COVID19 in the Madrid Pharmacy Association premises by a team of physicians, nurses and -for the first time in Spain by community pharmacists. However, as of June 2021, such involvement of pharmacists in limited to Madrid region only.

## Turkey

According to existing the legal framework, pharmacists are not currently authorised to provide vaccinations in Turkey. No action has been yet taken by the Turkish Ministry of Health to involve either hospital or community pharmacists in COVID-19 vaccination. COVID-19 vaccines are supplied by the Ministry of Health and sent to hospitals and family health centres. Priority groups (e.g. healthcare workers including community pharmacists and pharmacy staff) announced by the Ministry of Health could receive their COVID-19 vaccine by getting an appointment from hospitals and family health centres.

Pharmacists are currently contributing to the vaccination drive by providing education and counselling to the public. Current research organised by Turkish Pharmacists Association is exploring the perceptions of community pharmacists, general practitioners, and the public on pharmacists adopting a more active role in the vaccination programme. An online COVID-19 vaccination information platform was developed by the Ministry of Health to inform the public about recent updates and information about COVID-19 vaccines and vaccination programmes.

## United Kingdom (UK)

Health-related policy is devolved across the UK countries thereby allowing England, Scotland, Northern Ireland and Wales to have their own vaccination roll out strategies. However, across the UK, pharmacists have been in the forefront of the vaccination drive. Primary Care Network (PCN) and community pharmacy sites are actively participating in COVID-19 vaccinations alongside the hospitals and community vaccination centres (such as those located in football stadiums) [10, 11]. Currently pharmacists can either enter into a sub-contracting arrangement with the PCN and primary care general practice vaccination sites offering pharmacists, support staff or any other resources. In addition to this, pharmacists can host a vaccination site under a contractual agreement directly with NHS England. Pharmacists are allowed to use neighbouring facilities where their own premises are not suitable for vaccine administration. Members of the public can book a slot in the pharmacy directly through the NHS website. Indemnity arrangements are extended to cover pharmacy staff involved in the vaccination [33]. Pharmacies are remunerated £12.58 per vaccination administered through the NHS England remuneration scheme [34].

Emergency changes to the legislation were introduced which allowed preparation and administration of vaccination under the supervision of a pharmacist at a site which would not normally be classed as a registered pharmacy [10]. Patient Group Directions to support vaccine administration has been centrally made available at the national level.

## Discussion

Currently in Europe, diverse policies, practices and services exist regarding pharmacists’ involvement in COVID-19 vaccination. Countries such as Switzerland, UK and Ireland have measures in place to allow pharmacists to be directly involved in COVID-19 vaccine administration. However, their participation is also limited by issues around vaccine supply. These are expected to be addressed once vaccine demand is met by global manufacturing and supply. While current legislative hurdles are preventing pharmacists in many European countries from maximising their involvement in the roll-out of COVID-19 vaccines, a number of professional societies such as in Belgium, Portugal and Germany are advocating for changes to existing legislative provisions.

Given pharmacists’ diverse clinical expertise and the trust afforded to them by patients and the public, pharmacists have had a critical role during the initial phases of the pandemic. This includes the education and training of healthcare professional and patients, involvement in critical care of COVID-19 patients, participation in clinical trials and facilitation of routine clinical services during the pandemic. Therefore, involving pharmacists in administration of COVID-19 vaccinations could provide a vital way forward to allow mass vaccination in the shortest possible timeframe. Future studies should investigate patient acceptance and experiences of pharmacists’ involvement in the COVID-19 vaccination process to promote continued role expansion across European countries. There is also a need to review practices and policies in other European nations not represented in this paper and globally. Where pharmacists’ contribution remains limited to vaccine logistics, supply and preparation prior to administration, views of stakeholders including policy makers and other healthcare professionals should be sought to delegate further roles to pharmacists with a view to promoting changes in relevant legal frameworks to allow to administer vaccines. Improving pharmacists’ confidence, knowledge and skills and appropriate remunerations for their involvement in vaccinations are vital in ensuring effective vaccination programmes. Pharmacists’ participation may also require routine clinical services such as minor ailments management, drug history taking, dispensing of prescriptions, over-the-counter counselling be delegated to pharmacy technicians and support staff thereby freeing up pharmacists’ roles. Many countries including the UK have already since long implemented pharmacist independent prescribing services allowing pharmacists to prescribe medicines in their area of competence [35]. Involvement of pharmacists in COVID-19 vaccination roll-out seems to be a natural progression of their roles. To date, there are no reports to indicate any additional risks from the COVID-19 vaccine depending on the setting or personnel involved in COVID-19 vaccine administration. In addition, pharmacists’ participation in flu and travel vaccinations in many countries demonstrate their competency in related clinical roles.

## Conclusions

While some European countries are allowing pharmacists to administer vaccines, their roles are limited to logistics, reconstitution prior to vaccine administration and patient education in most countries. Pandemic mitigation strategies should incorporate further direct clinical roles of pharmacists including vaccine administration through legislative changes, additional training and accreditation of pharmacists where needed. Pharmacists’ ability and readiness to adjust current clinical services and innovate as demonstrated during the initial phase of the pandemic should be harnessed to mitigate repeat waves of the pandemic.
